# Cross-protective effect of a combined L5 plus L3 *Leishmania major* ribosomal protein based vaccine combined with a Th1 adjuvant in murine cutaneous and visceral leishmaniasis

**DOI:** 10.1186/1756-3305-7-3

**Published:** 2014-01-02

**Authors:** Laura Ramirez, Laura Corvo, Mariana C Duarte, Miguel A Chávez-Fumagalli, Diogo G Valadares, Diego M Santos, Camila I de Oliveira, Marta R Escutia, Carlos Alonso, Pedro Bonay, Carlos AP Tavares, Eduardo AF Coelho, Manuel Soto

**Affiliations:** 1 Centro de Biología Molecular Severo Ochoa (CSIC-UAM), Departamento de Biología Molecular. Nicolás Cabrera 1, Universidad Autónoma de Madrid, 28049 Madrid, Spain; 2 Programa de Pós-Graduação em Ciências da Saúde: Infectologia e Medicina Tropical, Faculdade de Medicina, Universidade Federal de Minas Gerais, Belo Horizonte, Minas Gerais, Brazil; 3 Departamento de Bioquímica e Imunologia, Instituto de Ciências Biológicas, Universidade Federal de Minas Gerais, Belo Horizonte, Minas Gerais, Brazil; 4 Centro de Pesquisas Gonçalo Moniz, FIOCRUZ, Salvador, Bahia, Brazil; 5 Research & Development Department, Laboratorios LETI S.L.u, 28760 Madrid, Spain; 6 Departamento de Patologia Clínica, COLTEC, Universidade Federal de Minas Gerais, Belo Horizonte, Minas Gerais, Brazil

**Keywords:** *Leishmania* parasites, L3 and L5 ribosomal proteins, BALB/c mice, Experimental infection, Vaccines, Visceral leishmaniasis, Cutaneous leishmaniasis

## Abstract

**Background:**

Two *Leishmania major* ribosomal proteins L3 (LmL3) and L5 (LmL5) have been described as protective molecules against cutaneous leishmaniasis due to infection with *L. major* and *Leishmania braziliensis* in BALB/c mice when immunized with a Th1 adjuvant (non-methylated CpG-oligodeoxynucleotides; CpG-ODN). In the present study we analyzed the cross-protective efficacy of an LmL3-LmL5-CpG ODN combined vaccine against infection with *Leishmania amazonensis* and *Leishmania chagasi (syn. Leishmania infantum)* the etiologic agents of different clinical forms of human leishmaniasis in South America.

**Methods:**

The combined vaccine was administered subcutaneously to BALB/c mice. After immunization the cellular and humoral responses elicited were analyzed. Mice were independently challenged with *L. amazonensis* and *L. chagasi*. The size of the cutaneous lesions caused by the infection with the first species, the parasite loads and the immune response in both infection models were analyzed nine weeks after challenge.

**Results:**

Mice vaccinated with the combined vaccine showed a Th1-like response against LmL3 and LmL5. Vaccinated mice were able to delay lesion development due to *L. amazonensis* infection and to control parasite loads in the site of infection. A reduction of the parasite burden in the lymph nodes draining the site of infection and in the liver and spleen was observed in the vaccinated mice after a subcutaneous infection with *L. chagasi*. In both models of infection, protection was correlated to parasite antigen-specific production of IFN-γ and down-regulation of parasite-mediated IL-4 and IL-10 responses.

**Conclusions:**

The data presented here demonstrate the potential use of *L. major* L3 and L5 recombinant ribosomal proteins for the development of vaccines against various *Leishmania* species.

## Background

Infection with different species from the genus *Leishmania* can cause a variety of clinical symptoms known globally as leishmaniasis [1]. Although some veterinary vaccines against leishmaniasis are now available [2-4] no vaccine has been developed for humans. During the last few years some advances in the development of vaccines against leishmaniasis have been carried out [5]. Given that first generation vaccines using crude parasite antigens were unable to induce protection [6] two main strategies have been explored for the development of anti-*Leishmania* vaccines. The first one employ live vaccines composed of molecularly modified attenuated parasites (leishmanization) to induce protective anti-*Leishmania* immune responses [7,8]. Alternatively, second generation vaccines are based on the use of parasite protein fractions [9,10] or individual parasite antigens [11]. Although some of these second generation vaccines are currently used in human clinical trials [12] the screening of new candidates will help to further increase the prophylactic efficacy of a *Leishmania* vaccine. It has been proposed that combination of different parasite antigens may help to attain a vaccine containing the most appropriate protective characteristics [5]. In addition, since multiple *Leishmania* species are distributed in the same or adjacent geographical regions [13] it would be desirable to develop vaccines containing candidates capable of inducing protection against the infection caused by various *Leishmania* species. One example of this situation is South America where the leishmaniasis disease ranges from visceral forms (VL) caused by *Leishmania chagasi* (syn. *Leishmania infantum*[14]) infection, to cutaneous forms (CL) caused by infection with different parasite species such as *Leishmania braziliensis*, *Leishmania. pifanoi* and *Leishmania amazonensis.* All these species can coexist in different geographical regions [13]. Thus, to be effective as a human vaccine against leishmaniasis its components should be shared by different parasite species and, prior to its use in humans, the protective efficacy of these candidates should be analyzed in different models of experimental leishmaniasis.

Examples of such vaccine preparations are those based on parasite ribosomal proteins. It has been demonstrated that a preparation of biochemically purified *Leishmania* ribosomal proteins (LRP) administered in combination with Th1 inducing adjuvants conferred protection against the challenge with different parasite species: *L. amazonensis* and *L. chagasi*[15] or *L. major* promastigotes [16]. Also, vaccinated and protected BALB/c mice were able to control the disease due to a secondary parasite challenge [17]. It was recently reported that two of the large subunit constituents of *L. major* ribosomes L3 or L5, expressed as recombinant proteins (LmL3 and LmL5) and administered independently or in combination (always in the presence of a Th1 adjuvant such as non-methylated CpG-oligodeoxynucleotides; CpG-ODN) were able to control the outcome of infection in an experimental model of American CL, namely BALB/c mice infected with *L. braziliensis*[18]. Globally, it was found that protection was associated with both, the induction of LRP-, LmL3- or LmL5-specific IFN-γ mediated responses and the control of the antigen dependent production of the susceptibility associated cytokines IL-10 and IL-4 [19].

The first objective of the study was to analyze the immunogenic properties of a vaccine combining the LmL3 and LmL5 recombinant proteins and CpG-ODN as adjuvant in BALB/c mice. The second objective was to study the combined vaccine prophylactic properties, challenging immunized mice with two different *Leishmania* species: *L. amazonensis* and *L. chagasi.* The potential mechanism of the combined vaccine-induced observed protection was also investigated and is consistent with the maintenance of the Th1-like response against the LmL3 and LmL5 antigens induced by vaccination after infection.

## Methods

### Antigens and adjuvant

Soluble *Leishmania* antigenic (SLA) extract was prepared from stationary-phase promastigotes of *L. major*, *L. chagasi* and *L. amazonensis* as previously described [20]. *L. major* ribosomal proteins (LRP) or mouse ribosomal proteins (MRP) were prepared from logarithmic-phase promastigotes of *L. major* and RAW 264.7 murine macrophage cells, respectively, as previously described in [10].

LmL3 and LmL5 recombinant proteins were over-expressed in *Escherichia coli* (M15 strain), purified under denaturing conditions onto Ni-nitrilotriacetic-acid-agarose columns (Qiagen, Hilden, Germany) and refolded on the affinity column, as described in [21]. Polymyxin-agarose columns (Sigma, St. Louis. MO, USA) were employed to remove residual endotoxin content (<10 ng of LPS per 1 mg of recombinant protein, measured by the Quantitative Chromogenic Limulus Amebocyte Assay QCL-1000 (BioWhittaker, MD, USA)).

Phosphorothioate-modified CpG-ODN (5′-TCAACGTTGA-3′ and 5′- GCTAGACGTTAGCGT-3′) were synthesized by Isogen Life Science B.V. (De Meern, The Netherlands) and employed for their capacity to induce Th1 responses in mice when immunized with different leishmanial antigenic preparations [16,22].

### Immunization, challenge infection, cutaneous lesion development and parasite quantitation

The Bioethical Committee of the Consejo Superior de Investigaciones Científicas (CEEA-11/046) and the Universidad Autónoma de Madrid (CEI 21–443) in Spain and the Animal Use Committee of the Federal University of Minas Gerais (CEUA; 047/2009) in Brazil approved the experimental. Mice (n = 4 or 5) were subcutaneously (s.c.) immunized in their left hind footpads with a mixture of the LmL3 and LmL5 recombinant proteins (6 μg each) plus 25 μg of each CpG-ODN (combined vaccine). As control groups, mice (n = 4 or 5) were inoculated with 25 μg of each CpG-ODN or with saline (PBS; vaccine diluent). Each group was boosted two and four weeks later with the same dose. For challenge, immunized mice were s.c. infected, into the right hind footpad, with 1 × 10^7^ stationary-phase promastigotes of *L. chagasi* (n = 4, per group) or with 1 × 10^6^ stationary-phase promastigotes of *L. amazonensis* (n = 4 per group). In mice infected with *L. amazonensis*, footpad swelling was measured with a metric caliper (the thickness of the left footpad minus thickness of the right footpad is shown). At week nine post-challenge all animals were sacrificed. For parasite load determination, the footpads of mice infected with *L. amazonensis* were taken and weighed before their individual processing. In addition the whole spleen, liver and the single popliteal lymph node draining the site of infection (DLN, right leg) of mice s.c. infected with *L. chagasi* were collected and independently processed as follows. Samples were mechanically homogenized in complete Schneider’s medium (Schneider’s medium (Sigma) supplemented with 20% heat-inactivated fetal bovine serum (FBS, Sigma), 20 mM L-glutamine, 200 U/ml penicillin, 100 μg/ml streptomycin and 50 μg/ml gentamicin) and filtered using a cell strainer (70-μm pore size). Each homogenized sample tissue was serially diluted in a 96-well flat-bottomed microtiter plate containing the same medium (in triplicates). The number of viable parasites (by mg of tissue for the footpads and by organ in the spleen, liver and DLN) was determined from the highest dilution at which promastigotes could be grown with up to 10 days of incubation at 25°C as previously described [23].

### Mice and parasites

Female BALB/c mice (6–8 weeks old) were purchased from Harlan (BCN, Spain) or from the Institute of Biological Sciences, ICB, Federal University of Minas Gerais (Belo Horizonte, Brazil). First, the immunization procedure was carried out using a total number of 15 mice (5 mice immunized with saline, 5 mice immunized with the adjuvant and 5 mice immunized with the combined vaccine). Mice were euthanized one month after the last immunization for the analysis of the immune response elicited by vaccination. Next, mice were immunized subcutaneously with the combined vaccine (n = 12), with the vaccine diluent (n = 12) or with the vaccine adjuvant (n = 12). One month after vaccination, 4 mice per group were euthanized to test the reproducibility of the vaccine induced response. The remaining animals were infected s.c. with *L. amazonensis* (n = 4 mice per group) or *L. chagasi* (n = 4 per group) to analyze the effect of vaccination in leishmaniasis progression. This last assay was reproduced using the same number of mice.

Regarding parasites, *L. major* clone V1 (MHOM/IL/80/Friedlin), *L. chagasi* (MOM/BR/1970/BH46) and *L. amazonensis* (IFLA/BR/1967/PH-8) parasites were grown at 25°C in complete Schneider’s medium.

### Cytokine production

Spleen cells obtained from each mouse were seeded and independently cultured in RPMI complete medium at 5 × 10^6^ cells per ml (RPMI medium (Sigma) supplemented with 10% heat-inactivated FBS, 20 mM L-glutamine, 200 U/ml penicillin, 100 μg/ml streptomycin and 50 μg/ml gentamicin) during 48 h at 37°C in 5% CO_2_ alone or with some of the next stimuli: recombinant LmL3 (12 μg/ml), recombinant LmL5 (12 μg/ml), SLA (from the indicated species, 12 μg/ml), LmLRP (12 μg/ml) and MRP (12 μg/ml). The release of IFN-γ, IL-10 and IL-4 was measured in culture supernatants by sandwich ELISA using monoclonal antibodies specific for mouse cytokines (capture and detection) provided in commercial kits (Pharmingen, San Diego, CA, USA), following the manufacturer’s instructions.

### Analysis of the humoral responses

Animals (n = 4 per group) were bled four weeks after the last immunization and nine weeks after challenge and the anti-LmL3-, anti-LmL5- or anti-SLA specific IgG1 and IgG2a antibodies present in the sera were measured by ELISA, as described elsewhere [20]. Briefly, 96-well plates (Becton Dickinson, Franklin Lakes, NJ, USA) were sensitized with the recombinant proteins or SLA (from the indicated species) at 10 μg/ml (each one) in PBS (100 μl/well) for 16 h at 4°C. Plates were blocked with PBS-10% bovine serum albumin at 37°C for 1 h and treated with 1/200 dilutions of mouse serum samples for 2 h at 37°C. Peroxidase-conjugated anti-mouse IgG1 or IgG2a isotype (Sigma) was diluted at 1:5,000 (for recombinant proteins) or 1:10,000 (for SLA) and added for 1 h at 37°C. Reactions were developed by incubation with H_2_O_2_ and O-phenylenediamine. Optical densities were read at 492 nanometers in a spectrophotometer (Molecular Devices, Spectra Max Plus, Concord, Canada).

### Statistical analysis

Statistical analysis with the vaccinated and infected mice was performed by a two-tailed Student’s *t*-test. Differences were considered significant when *P* < 0.05.

## Results and discussion

### Immunogenicity of the LmL3 + LmL5 + CpG-ODN combined vaccine in BALB/c mice

Since combination of different parasite protective antigens have been defined as an adequate strategy for *Leishmania* vaccine development [24,25] we decided to test a LmL3 + LmL5 + CpG-ODN combined vaccine based on the observation that both proteins were able to induce protection against murine CL due to *L. major* infection [19]. Moreover, administration of the CpG-ODN adjuvant combined with these antigens as single or combined vaccines induced a robust protection in mice against infection with a mixture of *L. braziliensis* stationary-phase promastigotes and insect vector saliva, while no protection was observed when animals were only treated with CpG-ODN [19]. However, the immune response elicited against the antigens by their co-administration was not analyzed in that work.

**Figure 1:**
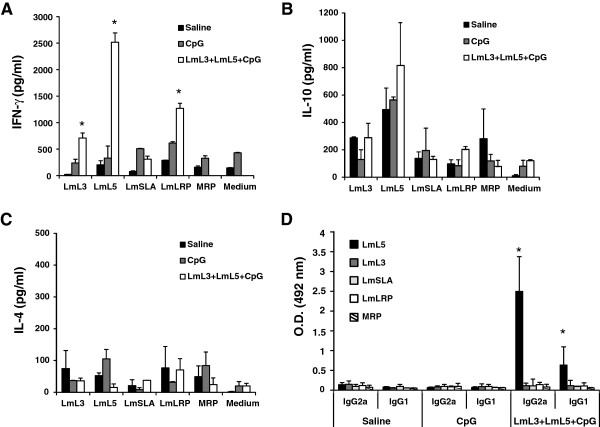
**Cellular and humoral responses in BALB/c mice immunized with the LmL3 + LmL5 + CpG-ODN combined vaccine.** Spleen cell cultures were established four weeks after the last vaccine dose and maintained for 48 h at 37°C, 5% CO_2_. As control, spleen cell cultures from mice immunized with the vaccine diluent (Saline) and the CpG-ODN adjuvant (CpG) were prepared at the same time. Splenocytes were grown without stimulus (medium; background control) or independently stimulated with the recombinant LmL3 or LmL5 proteins (at 12 μg/ml each one), with Soluble *L. major* Antigen (LmSLA), *L. major* Ribosomal Proteins (LmLRP) or Mouse Ribosomal Proteins (MRP) (at 12 μg/ml for each extract). **(A)** IFN-γ, **(B)** IL-10 and **(C)** IL-4 levels were assessed by ELISA in culture supernatants. Each bar represents the mean + standard deviation (SD) of data from five individual mice. * *P* < 0.05; significant cytokine production increase between LmL3 + LmL5 + CpG-ODN vaccinated mice and both control groups. **(D)** IgG1 and IgG2a antibodies against LmL3, LmL5, SLA, LRP and MRP were measured in serum samples from the three mice groups. Samples were obtained four weeks after the last vaccine dose and individually tested by ELISA at 1/200 dilution. Each bar represents the mean + SD of data from five individual mice. * *P* < 0.05; significant increase in the optical density between LmL3 + LmL5 + CpG-ODN vaccinated mice and both control groups. Data shown are representative of at least two independent experiments with similar results.

From an immunological point of view, we show that spleen cells from mice immunized with the combined vaccine were able to secrete LmL3- and LmL5-specific IFN-γ, since significantly higher levels of this cytokine were found in the culture supernatants after stimulation with these antigens, when compared with the culture supernatants established from control mice, namely saline (*P* = 0.0006 for LmL3 and *P* = 0.0002 for LmL5) and CpG-ODN groups (*P* = 0.0002 for both antigens) (Figure 1A). The presence of the CpG-ODN, an agonist of the TLR9 [26] that confers a Th1-related long-term immunity and protection when combined with different leishmanial antigens [22] was essential for the stimulation of the antigen specific IFN-γ response, since low levels of this cytokine were produced when the LmL3 + LmL5 proteins were co-administered in the absence of the adjuvant (not shown). The IFN-γ mediated response was comparable to that induced when each one of the antigens was administered in the presence of the same adjuvant [19]. The LmL3 and LmL5 combined vaccine also induced similar patterns of IL-10 and IL-4 secretion than single based vaccines [19]. Spleen cells from vaccinated mice showed a slightly higher production of IL-10 than control mice groups when stimulated with LmL5, although the differences were not significant (Figure 1B). Very low similar levels of IL-4 were detected in vaccinated and control groups after stimulation with both recombinant proteins (Figure 1C). In addition, vaccinated mice showed a specific anti-LmL5 humoral response that was predominantly of the IgG2a isotype (*P* = 0.01 compared with saline and CpG-ODN groups) although IgG1 antibodies specific for this antigen were also observed in the sera from immunized mice (*P* = 0.03 compared with saline and CpG-ODN groups) (Figure 1D). It can be concluded that the vaccine combining LmL3, LmL5 and CpG-ODN induced a predominant Th1-like response for both antigens of different magnitude, stronger for LmL5 than for LmL3, because of the robust production of LmL5-specific IFN-γ and by the presence of anti-LmL5 IgG2a antibodies, markers of Th1-type responses [27]. The immune response elicited against the recombinant proteins was correlated with a production of IFN-γ when *L. major* ribosomes (LmLRP) were employed for *in vitro* stimulation (Figure 1A; LmLRP). The lack of LmSLA-specific production of cytokines (Figure 1A-C; LmSLA) may be related to the lower contents of ribosomal proteins in a total parasite extract when compared to the LmLRP preparation.

*Leishmania* LmL3 and LmL5 proteins were selected for a cross-protection analysis due to their high degree of conservation among different *Leishmania* species [19]. On the other hand, and regarding host counterparts, *L. major* LmL3 and LmL5 showed lower identity and similarity scores with respect to their mouse homologs: *Mus musculus* L3 (NCBI: NP_038790.2), identity: 59.4%, similarity: 74.0%; *Mus musculus* L5 (NCBI: NP_000960.2), identity: 53.4%, similarity: 68.5%. Remarkably, no humoral or cellular responses against host ribosomal proteins were observed in vaccinated mice (Figure 1A-D; MRP). This lack of immune cross-reactivity between parasite and host intracellular proteins belonging to conserved families was also observed for other intracellular antigens, such as histones and heat shock proteins in infected individuals [28]. This observation has been related to the location of B and T cell epitopes in the most divergent regions of these parasite proteins [28-30].

### Effects of vaccination in the development of CL due to *L. amazonensis* and VL due to *L. chagasi* in BALB/c mice

**Figure 2:**
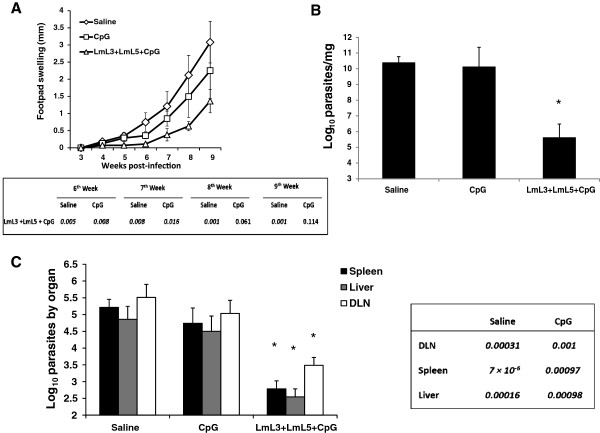
**Effect of the vaccination in murine leishmaniasis due to *L. amazonensis *or *L. chagasi *infection*.*** Mice (four per group) immunized with vaccine diluent (Saline) with CpG-ODN adjuvant (CpG) or with the recombinant vaccine (LmL3 + LmL5 + CpG) were independently challenged subcutaneously in the left footpad with 1 × 10^6^ stationary-phase promastigotes of *L. amazonensis***(A-B)** or 1 × 10^7^ stationary-phase promastigotes of *L. chagasi***(C)**. **(A)** Lesion development was monitored weekly during nine weeks in mice infected with *L. amazonensis*. The mean ± standard deviation (SD) of the footpad swelling is given as the difference of thickness between the infected and the uninfected contralateral footpad. *P* values (saline immunized versus vaccinated or CpG-ODN immunized versus vaccinated) are included in the table below the graph. **(B)** Parasite burden was determined in the infected footpads (parasites per mg) nine weeks after infection with *L. amazonensis*. Mean + SD is shown. * *P* < 0.05 indicates significant decrease in the footpad parasite burdens between vaccinated and both control groups. **(C)** Parasite burdens (parasites per organ) in the spleen, in the liver and in the popliteal lymph node draining the infected footpad (DLN) after *L. chagasi* infection. Mean + SD of four mice in each group is shown. *P* values (saline immunized versus vaccinated or CpG-ODN immunized versus vaccinated) are included in the table at the right of the graph. Data shown are representative of two independent experiments with similar results.

Since the development of vaccines against leishmaniasis requires the definition of potential candidates capable of inducing protective responses against different *Leishmania* species [12], we evaluated the protective efficacy of the combined vaccine (LmL3 + LmL5 + CpG-ODN) in two different forms of murine leishmaniasis. The first one was a model of infection with a high dose of *L. amazonensis* stationary promastigotes in the footpad of BALB/c mice. This highly pathogenic species is able to cause different forms of American Tegumentary leishmaniasis (ATL) and VL in humans and a severe CL in mice [31,32]. Mice immunized with the combined vaccine showed a reduction in the lesion size when compared with control mice groups immunized with saline or with the adjuvant alone (Figure 2A). Differences were significant until nine weeks after infection when compared with saline and until seven weeks when compared with CpG-ODN group (Figure 2A). At the end of the assay (nine weeks after challenge), the parasite burdens found in the infected footpads were lower in vaccinated mice than in both controls groups (Figure 2B).

The decrease in parasite loads in the infected footpads was statistically significant when compared with both groups (*P* = 1.6 × 10^-5^ and *P* = 4.5 × 10^-5^ for saline and CpG-ODN groups, respectively). It was concluded that although the administration of the adjuvant alone could have a slight influence on the outcome of infection, the administration of the combined vaccine induced a delay of the progressive disease due to the *L. amazonensis* challenge. The BALB/c-*L. amazonensis* model of infection has been employed for a limited number of antigenic preparations including some antigens described as protective in other forms of the disease like the LACK protein or the amastigote A2 antigen (reviewed in [11]) and the *Leishmania* P4 nuclease, a protein immunogenic in humans infected with *L. major*[33] and related to protection against experimental murine *L. pifanoi* infection [34]. Among them, the most protective formulation was based on defined antigens administered in combination with IL-12: P4 nuclease [35] or the A2 protein [20], since no footpad swelling was observed after *L. amazonensis* challenge. Although it is difficult to establish a direct comparison between different vaccine candidates (due to the use of different parasite strains, number of parasite in the inoculum, adjuvants employed, etc.), the combined vaccine assayed here seems to be inducing weaker protection than vaccines that employ IL-12 as adjuvant, a cytokine that plays a central role in promoting Th1 responses and cell-mediated immunity [36]. The effects of the combined vaccine are more comparable with other vaccines based on parasite total proteins [37-39], antigenic extracts [38-40] including LRP + saponin [15] and some DNA vaccines based on the A2 [41] or LACK [39] proteins, that induced a delay in the footpad swelling. Of interest, the 4.5-log reduction observed in the footpad parasite loads of mice vaccinated with the LmL3 + LmL5 + CpG-ODN preparation with respect to both control groups (Figure 2B) was comparable in magnitude with parasite burden differences observed in mice vaccinated with the most protective formulations and their controls [20,35,41]. Moreover, it should be taken into account that some antigen-based vaccines, such as LACK combined with IL-12 [20] or a DNA vaccine based on a Nucleoside Hidrolase [41] an antigen that induces partial protection against other *Leishmania* species [34], were unable to control murine CL due to *L. amazonensis* infection. In addition, some parasite serine-proteases were able to exacerbate the *L. amazonensis* related disease when immunized as a prophylactic preparation alone or combined with saponin [42].

A great number of different molecules have been tested as second generation vaccines in murine models of VL infection [43]. Most of the tested antigens were studied using the intravenous route of infection that guarantee the induction of VL but could undervalue the potential efficacy of some vaccines [44]. We decided to subcutaneously challenge *L. chagasi* in the footpad of BALB/c mice because this model has been accepted as an optimal screening tool to analyze protective antigens [45] and has been previously employed to test the immunoprophylactic properties of different parasite components [46,47], including the combination of LRP and saponin [15]. As it is shown in Figure 2C the *L. chagasi* challenge resulted in parasite active infection with the presence of parasites in the DLN (in the absence of footpad swelling) but also in the spleen and in the liver, internal organs involved in parasite replication in murine VL [48]. Nine weeks after infection, mice immunized with LmL3 + LmL5 + CpG-ODN showed significantly lower parasite burdens than both control groups in the three analyzed organ locations (Figure 2C). The decrease of parasite loads in the vaccinated mice was more evident in the internal organs (2.5-log reduction in the liver and in the spleen when compared with saline group and 2-log reduction in the liver and in the spleen when compared with the CpG-ODN group). A significant decrease in the parasite burdens of the lymph node draining the infected footpad (2-log and 1.5-log reduction when compared with saline and CpG-ODN groups, respectively) was also observed. As it also occurred after *L. amazonensis* challenge (the present study) and other cutaneous species (*L. major* and *L. braziliensis*[19]) vaccinated mice were able to control the replication of different *Leishmania* species, allowing the conclusion that LmL3 and LmL5 based vaccines will fit the requirements to a pan-*Leishmania* vaccine.

### Immunological parameters associated with protection

To determine the immunological parameters of protection, the production of different cytokines in the supernatants of spleen cell cultures, established from the different groups of mice and stimulated with different antigenic preparations was analyzed.

**Figure 3:**
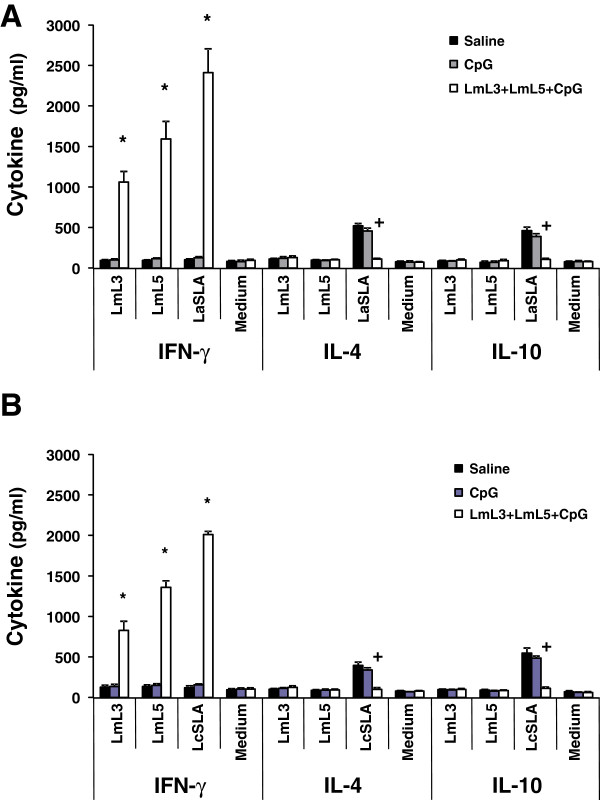
**Production of cytokines by spleen cells of vaccinated mice after *Leishmania *infection*.*** Mice (four per group) immunized with vaccine diluent (Saline), with CpG-ODN adjuvant (CpG) or with the recombinant vaccine (LmL3 + LmL5 + CpG) were independently challenged subcutaneously in the left footpad with 1 × 10^6^ stationary- phase promastigotes of *L. amazonensis***(A)** or 1 × 10^7^ stationary-phase promastigotes of *L. chagasi***(B)**. Nine weeks after infection, spleen cell cultured suspensions were non-stimulated (Medium; background control) or separately stimulated with recombinant LmL3 or LmL5 (12 μg/ml each one) or with SLA from *L. amazonensis* (LaSLA; A) or *L. chagasi* (LcSLA, B) at 12 μg/ml each one for 48 h at 37°C, 5% CO_2_. IFN-γ , IL-4 and IL-10 levels were measured in culture supernatants by ELISA. Mean + standard deviation (SD) of cytokine levels determined in four individual mice per group is shown; *, *P* < 0.05 indicates significant cytokine production increase between LmL3 + LmL5 + CpG-ODN vaccinated mice and both control groups; +, *P* < 0.05 indicates significant cytokine production decrease between LmL3 + LmL5 + CpG-ODN vaccinated mice and both control groups. Data shown are representative of two independent experiments with similar results.

**Figure 4:**
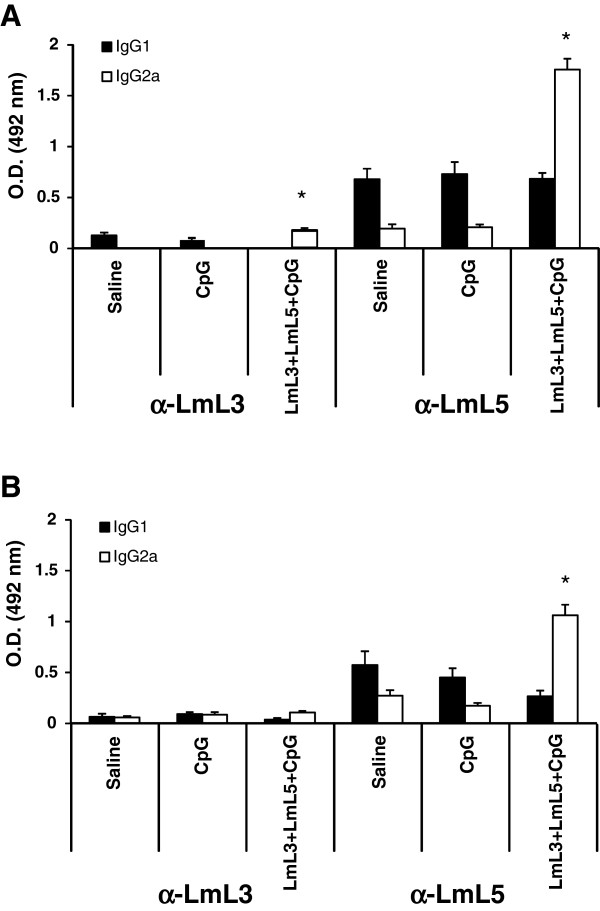
**Humoral response against the recombinant antigens following infection with *L. amazonensis *(A) or *L. chagasi *(B).** Nine weeks after parasite challenge vaccinated and control mice (n = 4 per group) were bled and sera were individually tested by ELISA for specific anti-LmL3 and anti-LmL5 antibody responses of both IgG1 and IgG2a isotype at 1/200 dilution. Each bar represents the mean + standard deviation (SD) of data from four individual mice. * *P* < 0.05 indicates significant increase in the optical density between LmL3 + LmL5 + CpG-ODN vaccinated mice and both control groups. Data shown are representative of two independent experiments with similar results.

First, the post-infection response against the antigens composing the vaccine was examined. Vaccinated and infected mice showed an IFN-γ LmL3- and LmL5-specific production that was absent in the infected control groups. A significant increase in the level of this cytokine in the supernatant of vaccinated versus saline and CpG spleen cells cultures was observed after stimulation with LmL3: *P* = 0.0006 for both antigens in *L. amazonensis* infected mice (Figure 3A) and *P* = 0.0008 and *P* = 0.0007, respectively, in *L. chagasi* infected mice (Figure 3B). Similarly, stimulation with LmL5 resulted in the secretion of higher levels of IFN-γ in vaccinated than in saline and CpG control mice groups: *P* = 0.0008 for both antigens in *L. amazonensis* infected mice (Figure 3A) and *P* = 2 × 10^-5^ and *P* = 3 × 10^-5^, respectively, in *L. chagasi* infected mice (Figure 3B). We did not detect antigen specific IL-4 or IL-10 production after stimulation with the recombinant LmL3 or LmL5 proteins in the vaccinated and infected mice. Thus, it was concluded that after challenge the Th1-like response elicited against the LmL3 and LmL5 ribosomal proteins by the vaccine was maintained. Following infection, cellular responses elicited against LmL5 were higher than those detected against LmL3. The different immunogenic properties of the two recombinant proteins were also observed in the humoral responses elicited against both proteins in the infected mice. In both mouse models, *L. amazonensis* vaccinated and infected mice (Figure 4A) or *L. chagasi* vaccinated and infected mice (Figure 4B) the reactivity against LmL5 was stronger than against LmL3, as also occurred in mice vaccinated with these proteins after *L. major* infection [19]. In the *L. amazonensis* model higher IgG2a anti-LmL3 and anti-LmL5 antibody reactivity were observed in vaccinated than in saline mice groups (*P* = 0.0063 and P = 0.0006, respectively) or in CpG-ODN immunized mice (*P* = 0.002 and P = 0.001, respectively) (Figure 4A). After *L. chagasi* infection only the IgG2a anti-LmL5 antibodies showed higher reactivity in vaccinated than in control mice (*P* = 0.002 saline and P = 0.003 CpG-ODN) (Figure 4B).

**Figure 5:**
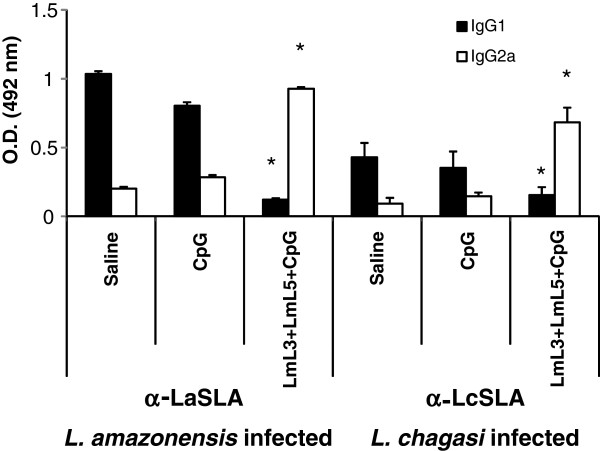
**Humoral response against soluble parasite antigens (SLA) antigens following infection with *L. amazonensis *or *L. chagasi*.** Nine weeks after parasite challenge vaccinated and control mice (n = 4 per group) were bled and sera were individually tested by ELISA for specific anti-SLA antibody responses of both IgG1 and IgG2a isotypes at 1/200 dilution. Each bar represents the mean + standard deviation (SD) of data from four individual mice. * *P* < 0.05 indicates significant changes in the optical density between LmL3 + LmL5 + CpG-ODN vaccinated mice and both control groups. Data shown are representative of two independent experiments with similar results.

The systemic cellular response in infected BALB/c mice against *L. amazonensis* parasite total proteins was analyzed by LaSLA (prepared from this parasite specie) stimulation of spleen cell cultures established from saline and CpG-ODN control groups. SLA specifically induced the secretion of IL-4 and IL-10 production in the absence of IFN-γ production in both infected control groups (Figure 3A). This low T- cell response also reported for other experimental infections performed with *L. amazonensis*[15,20,41,49] can negatively affect the activation of the effector functions of macrophages for destroying the intracellular amastigotes [31,50]. On the contrary, after infection vaccinated mice mounted a LaSLA-specific IFN-γ mediated response (vaccinated mice versus saline [*P* = 0.0005] or CpG-ODN [*P* = 0.0006] groups) (Figure 3A), that may be activating the macrophages for destruction of parasites at the infection site, resulting in the observed decrease of the parasite load shown in Figure 2B. As reported here, induction of parasite specific IFN-γ responses by vaccination was also related to protection induced by parasite lysates [15,38,39] or single parasite proteins based vaccines [35,39]. In addition, a decrease in the LaSLA-mediated IL-4 and IL-10 responses was observed in the vaccinated and infected mice (Figure 3A). A comparison between saline and CpG-ODN controls and protected mice revealed that spleen cells from vaccinated mice produced significantly lower amounts of IL-4 (*P* = 2 × 10^-5^ and *P* = 0.0002, respectively) and IL-10 (*P* = 0.002 and *P* = 0.0001, respectively). A decrease in IL-10 was correlated with protection induced by the P4 Hidrolase protein + IL-12 vaccine [35] and a decrease of IL-4 and IL-10 parasite-specific mediated responses was also observed in mice protected by the administration of LRP + saponin [15], although the exact role of these cytokines in the development of lesions due to *L. amazonensis* is not fully understood [31]. The humoral responses elicited against total parasite proteins were in accordance with the cellular responses induced by infection, since anti-LaSLA antibodies found in the vaccinated mice were mainly of the IgG2a isotype, being the anti-LaSLA reactivity of the IgG1 isotype antibodies significantly lower than those detected in both control groups (*P* = 0.0006 versus saline and *P* = 0.0007 versus CpG-ODN, respectively) (Figure 5).

Similarly, when the cellular responses elicited in BALB/c mice infected with *L. chagasi* against the LcSLA (using extracts prepared from this parasite specie) were analyzed, a LcSLA-specific production of IFN-γ was detected in the vaccinated mice that was absent in saline (*P* = 5 × 10^-9^) or CpG-ODN (*P* = 4 × 10^-7^) groups (Figure 3B). This response was correlated to the predominant presence of anti-LcSLA IgG2a antibodies in the sera from vaccinated and infected mice (Figure 5). Since IFN-γ dependent activation of infected macrophages for production of nitric oxide is necessary for *Leishmania* intracellular killing [51] this cytokine has been considered one of the main factors implicated in the acquired immunity against infection with viscerotropic *Leishmania* species [15,41,52,53]. IL-10 parasite mediated responses are critical for VL progression [54], since BALB/c mice lacking the gene for IL-10 [55] or BALB/c mice treated with an anti-IL-10 receptor antibody [56] are resistant to infection. In accordance, mice vaccinated with the LmL3 + LmL5 + CpG-ODN combined vaccines showed a specific decrease in the LcSLA-mediated IL-10 (*P* = 0.0004 for saline and *P* = 8 × 10^-7^ for CpG-ODN) (Figure 3B) and also a controlled production of IL-4 specific for the parasite antigens (*P* = 7 × 10^-5^ for saline and *P* = 2 × 10^-5^ for CpG-ODN) (Figure 3B) correlated to the presence of low levels of anti-LcSLA IgG1 reacting antibodies (Figure 5). Although the implication of IL-4 mediated responses in murine VL progression has not been clearly demonstrated, various reports have correlated the induction of protection against a subcutaneous challenge with *L. chagasi* to the control of *Leishmania*-specific IL-4 mediated responses [15,41,46,47].

## Conclusions

The present study indicated that the administration of a vaccine based on the combination of the recombinant versions of the *L. major* ribosomal proteins L3 and L5 in the presence of a Th1 adjuvant (CpG-ODN) conferred cross-protection in BALB/c mice against subcutaneous infection with two different *Leishmania* species: *L. amazonensis* and *L. chagasi*. After vaccination, mice showed an LmL3, LmL5 and LRP (*Leishmania* ribosomal proteins) Th1-like response as shown by the production of IFN-γ specific for these antigens, in the absence of IL-10 or IL-4-specific responses. In spite of the conserved nature of the ribosomal proteins, vaccinated mice did not show cellular and humoral responses against the ribosomal protein of the vertebrate host (MRP extract). The immune response against LmL3 and LmL5 elicited by the combined vaccine was maintained after infection in the vaccinated and protected mice. Protection was also correlated with the induction of parasite dependent IFN-γ responses and with the down-regulation of parasite dependent IL-4 and IL-10 responses. Since LmL3 and LmL5 based vaccines were able to induce protection against different *Leishmania* species in BALB/c mice (*L. amazonensis* and *L. chagasi*; this work) and other cutaneous species such as *L. major* and *L. braziliensis*[19] we may conclude that these antigens could play a relevant role as components of a pan-*Leishmania* vaccine.

## Competing interests

The authors declare that they have no competing interests.

## Authors’ contributions

LR, LC, MCD, MACF, DGV, MRE and MS carried out the experimental procedures. MACF, DMS, CIO, MRE, CA, PB, CAPT, EAFC and MS conceived the research, contributed with data analysis and revised the manuscript. EAFC and MS wrote the manuscript. All authors read and approved the final version of the manuscript.
